# A model of the extent and distribution of woody linear features in rural Great Britain

**DOI:** 10.1002/ece3.2607

**Published:** 2016-11-22

**Authors:** Paul Scholefield, Dan Morton, Clare Rowland, Peter Henrys, David Howard, Lisa Norton

**Affiliations:** ^1^ Centre for Ecology and HydrologyLancaster Environment CentreBailriggLancashireUK

**Keywords:** classification, Countryside Survey, Land Cover Map 2007, landscape ecology, linear hedgerow network, rural planning

## Abstract

Hedges and lines of trees (woody linear features) are important boundaries that connect and enclose habitats, buffer the effects of land management, and enhance biodiversity in increasingly impoverished landscapes. Despite their acknowledged importance in the wider countryside, they are usually not considered in models of landscape function due to their linear nature and the difficulties of acquiring relevant data about their character, extent, and location. We present a model which uses national datasets to describe the distribution of woody linear features along boundaries in Great Britain. The method can be applied for other boundary types and in other locations around the world across a range of spatial scales where different types of linear feature can be separated using characteristics such as height or width. Satellite‐derived Land Cover Map 2007 (LCM2007) provided the spatial framework for locating linear features and was used to screen out areas unsuitable for their occurrence, that is, offshore, urban, and forest areas. Similarly, Ordnance Survey Land‐Form PANORAMA®, a digital terrain model, was used to screen out where they do not occur. The presence of woody linear features on boundaries was modelled using attributes from a canopy height dataset obtained by subtracting a digital terrain map (DTM) from a digital surface model (DSM). The performance of the model was evaluated against existing woody linear feature data in Countryside Survey across a range of scales. The results indicate that, despite some underestimation, this simple approach may provide valuable information on the extents and locations of woody linear features in the countryside at both local and national scales.

## 1 Introduction

Man‐made linear features marking boundaries are an integral part of landscapes throughout temperate regions (Barr and Petit, 2001). They are made of a range of different components including stone (walls and banks), vegetation (hedges, lines of trees, and grass strips), earth (banks), water (dykes), and wood or wire (fences). When woody linear features consisting of trees, shrubs, and bushes are regularly cut and laid, they can be defined as “managed hedges” (hereafter referred to as hedges) and are particularly widespread and ecologically important landscape features in farmed habitats (Baudry, Bunce, & Burel, 2000). Hedges were originally used to define or enclose fields making them stock‐proof, and standards or lines of trees within them were important to demarcate ownership boundaries. More recently, with the availability of relatively low‐cost and low‐maintenance fencing, land owners are putting much less effort into establishing and maintaining hedges (Antoine, 2001). However, a recent review investigating the potential importance of hedges to a range of ecosystem services (ES) at landscape scales indicated that they are not merely artifacts of previous management systems but may play a vital role in delivering services (Wolton, Pollard, Goodwin, & Norton, 2014) even in quite unexpected ways. For example, they have been shown to reduce the incidence of bovine tuberculosis in British cattle herds in high‐prevalence regions (Winkler & Mathews, 2015).

The multiple roles which hedges play in the supply of ES include (1) provision: food (sloes, berries, fungi, etc.) and firewood (Wolton, Pollard, et al., 2014); (2) regulation: modification of the microclimate in and around field systems, reduction of soil erosion by wind (Sanchez, Lassaletta, McCollin, & Bunce, 2010), carbon capture and storage in growing woody material and in litter (e.g., extensive linear networks, such as the bocage networks in France, contain considerable sequestered carbon (Robertson, Marshall, Slingsby, & Newman, 2012), restriction of the movement of agricultural livestock, and retention of water and sediment through their role as barriers to soil erosion and in the absorption and storage of water (Gascuel‐Odoux et al., 2011; Jongman & Bunce, 2009; Thomas, Ghazavi, Merot, & Granier, 2012; Van der Zanden, Verburg, & Mücher, 2013). In addition, certain species are also associated with key regulatory functions (see below); (3) supporting: soil creation, water and nutrient cycling, and species distribution networks (Thomas et al. 2008); (4) cultural: esthetics—hedges are included in definitions of English National Character Areas (Natural England 2014); (5) recreation—hedges support game species such as pheasant and attract wildlife, birds in particular, for enthusiasts to watch and enjoy (Hinsley & Bellamy, 2000); and (6) ownership—marking boundaries between different groups and owners. Hedges are recognized as being particularly important for biodiversity, and their value as semi‐natural habitats spanning increasingly ecologically impoverished agricultural landscapes is widely recognized (see Dainese, Montecchiari, Sitzia, Sigura, & Marini, 2016 and Morelli, 2013). Both the herbaceous flora which grows under and beside the woody shrubs (Roy & de Blois, 2008; Smart, Bunce, Firbank, & Coward, 2002) and the woody vegetation which forms the hedge provide important species and structural heterogeneity as well as providing connectivity between semi‐natural habitat components (Batary, Kovacs‐Hostyanszki, Fischer, Tscharntke, & Holzschuh, 2012; Roy & de Blois, 2008; Russ, Briffa, & Montgomery, 2003; Staley et al., 2012). By providing a refuge for a wide range of taxa effectively eliminated from fields as a result of agricultural improvement (Smart et al., 2006), woody linear features help to maintain functioning agro‐ecosystems in which predators of crop pests, pollinators, and pollen‐producing species all play their roles (Pocock, Evans, & Memmott, 2012; Barr and Petit, 2001; Baudry et al., 2000).

Despite the role that hedges may play in the delivery of services in the wider countryside, work investigating ES delivery at landscape scales (e.g., Burkhard et al. 2014) tends to ignore the contribution of hedgerows (and other linear features). Although boundary and linear features are defined as a Broad Habitat (part of a framework classification for 37 habitat types across the whole of the UK by JNCC, see Jackson, 2000), most researchers focus on the areal features within a landscape rather than on their borders and perimeters; consequently, there is a lack of spatial data detailing the types and locations of linear features across broad spatial scales.

The effective management of our natural resources for the future is dependent upon data describing its extent and condition (MEA, 2005). It can be monitored at any number of scales, but to understand resource management at a national level, it is important to have access to national data such as those used in the National Ecosystem Assessment (UK National Ecosystem Assessment 2011). Attempts to quantify the extent of boundary linear features at national scales are rare. One method, used in the Countryside Survey (CS), is stratified random sampling which used field survey to provide national statistics of the extent of the different linear features. CS used detailed field mapping of the extent and condition of linear features in nationally representative sample of 1‐km squares (Norton et al., 2012; Petit, Stuart, Gillespie, & Barr, 2003). Repeat surveys of the same squares make it possible to understand patterns of change in length and condition of hedges and lines of trees with recent results indicating declines in managed hedgerows as they decay into lines of trees (Norton et al., 2012). While estimates based on the same approach over time provide useful indices of change for policy makers and essential information for reporting, they do not provide valuable location‐specific information, except for in the actual squares in which CS takes place (those data remain confidential).

Field mapping of hedgerows at a national scale would be both expensive and time‐consuming; a potential alternative is the use of remote‐sensed or satellite data (Kerr & Ostrovsky, 2003). However, the spatial resolution of large‐scale remote‐sensed data makes an assessment of linear features more technically challenging than for elements such as land cover. The UK Land Cover Map (LCM2007; Morton et al., 2011) uses low‐resolution (25 m) thematic LandSat imagery interpreted as Broad Habitats, but does not identify the Boundaries and linear features Broad Habitat (Jackson, 2000). Previous attempts to map hedges from satellite imagery have led to generalized maps, for example, the French national hedgerow density maps, as developed by the L'Inventaire Forestier National (IFN) or more detailed regional hedgerow mapping (Vannier & Hubert‐Moy, 2008). Standard aerial photography and LiDAR (Light Detection And Ranging) offer better solutions (Black, Green, Mullooley, & Poveda, 2010), but for any region of moderate size are currently made difficult owing to the expense of data capture and magnitude of material to be stored and analyzed.

The work described here demonstrates for the first time a national coverage of linear features and builds on work reported in Scholefield, Norton, Rowland, Morton, and Henrys (2012) where the linear network was created by converting the LCM 2007 area framework (Smith & Fuller, 2001) to field boundaries. Great Britain (GB) is used as a case study to produce a predictive model of both woody linear features and other linear features which is then validated against existing Countryside Survey data at 1‐km square, land class (Bunce, Barr, Gillespie, & Howard, 1996) and national scales, although this approach could easily be applied elsewhere provided a linear network and a canopy height dataset are available. The model uses two key national datasets: (1) the LCM2007 spatial framework—based on that of the Ordnance Survey MasterMap (OSMM) topography layer which provides robust polygon boundaries for GB; and (2) the NEXTMap® Britain DSM series (hereafter referred to as NEXTMap), which provides digital terrain mapping for the UK land surface, indicating the height of features and land parcels above ground height. NEXTMap data are at relatively coarse resolution (5 m), but coverage for GB is comprehensive.

## 2 Methods

### 2.1 Model construction

The method used a simple classification of the attributes of each linear feature within a linear framework, carried out in ArcMap 10.3 (ESRI, 2015). Features within the framework were attributed from digital surface datasets, and then classified as hedges or other features based upon specific criteria determined from field survey data. First, nonsuitable areas were masked out where woody linear features were unlikely to be found or where it would be impossible to detect them, that is, where land was higher than 350 m, urban, wooded or in a coastal tide‐washed area. The network of boundaries or linear spatial framework was derived from LCM2007 which, in turn, drew its structure from OSMM. The boundary height information was calculated by subtracting the NEXTMap DTM that describes the altitude at ground level from the NEXTMap canopy surface model (DSM) that describes the altitude at the top of vegetative canopies for each line. Boundaries with woody linear features were identified from this calculated height data using thresholds for different vegetation height attributes for a given length of boundary, namely minimum vegetation height −0.13 m (accounting for the presence of a ditch adjacent to the woody feature) to maximum vegetation height 58 m (the maximum height for a tree in GB) and mean vegetation height 0.58 m (accounting for gappy features). These thresholds were therefore selected to enable differentiation between woody and other types of linear features. These values were selected by iteratively searching through these three height values to find the best fit to the CS2007 national estimates of woody linear feature length. The inputs were varied sequentially by 0.01‐m increments. The estimates were calculated for each survey square and then weighted for each stratum or land class in the survey. The figures were finally totalled across all land classes and compared to the estimates both for each land class from CS2007 and for the total length of woody linear features.

The datasets and modeling approaches are described in detail below. The model was validated against CS data at different spatial scales to provide information on its performance, as described.

### 2.2 Data inputs and feature attribution

A linear spatial framework formed the basis of the model. The Ordnance Survey Mastermap (OSMM) topography layer provides a detailed cartographic view of the landscape including individual buildings, point features, transport infrastructure, field boundaries, and areas of land. OSMM polygon objects (100 million) were used to create the spatial framework for LCM2007. As the spatial resolution of OSMM is greater than that used for LCM (which uses 20 m × 20 m pixel satellite data), the OSMM was spatially generalized, removing unnecessary detail while retaining relevant information on the location of boundaries (Morton et al., 2011). These data were then converted from a polygon format to a vector format, and the vectors split at intersections in order to yield a linear framework suitable for individual feature attribution from raster datasets (e.g., NEXTMap).

Surface relief information was obtained from the NEXTMap dataset, which was chosen as it has a comprehensive coverage of GB. NEXTMap includes both a DTM and a DSM, which were originally produced by Intermap Technologies in 2007, the same year as the Countryside Survey 2007. Data were generated by airborne survey using synthetic aperture radar (Carey et al., 2008) (SAR), and single‐pass interferometry (IfSAR; Chiverrell, Thomas, & Foster, 2008). NEXTMap digital elevation data were collected at a flight height of approximately 6,500 m; the data were supplied at a 5‐m resolution.

A spatial mask was necessary in order to filter or areas considered outside of hedgerow areas. NEXTMap 5‐m data were filtered using LCM 2007 (which describes land cover across GB in 2007) to remove *Built up*,* Woodland*,* Littoral,* and *Sub‐littoral* Broad Habitats. PANORAMA data (a gridded DTM with 50 m postspacing) were used to exclude all areas above 300‐m altitude by setting all canopy height data in these areas to zero. This coarser product was used for screening (OS Land‐Form PANORAMA in preference to NEXTMap) in order to generate a mode generalized surface to limit processing time. Canopy heights in wooded areas are such that they would mask the existence of woody linear features (hence the model, like CS, focused on rural areas and excluding hedges bounding or penetrating woodland). Littoral and sublittoral zones and land above 300‐m altitude were considered unlikely locations for woody linear features; hedgerows have not been recorded in CS locations with these characteristics. The resulting 5‐m resolution dataset was used in the model.

### 2.3 Model evaluation

The model was evaluated by comparing the model results at three scales, National (GB), GB land class and at the 1‐km resolution—these data were used as a “truth” to test the effectiveness of the model in terms of both the lengths and the spatial locations of woody linear features. The national estimates were generated for CS by summing the unweighted estimates of lengths from sample squares for all land classes. (Brown et al., 2014) ITE land classes result from a statistically generated stratification of all 1‐km squares across GB based on physical variables describing climate, altitude, morphology, geology, and some human geography (Bunce, Barr, Clarke, Howard, & Lane, 1996; Bunce, Barr, Gillespie, et al., 1996). Each land class consists of areas with a similar range of environmental characteristics. Sample squares for Countryside Survey are drawn by land class at random from 1‐km squares located on a 15‐km grid, to give a distributed stratified random sample of the GB countryside. For the 1‐km‐square‐level field data from CS2007 (Carey et al., 2008), land cover and ecological data were collected for a stratified distributed sample of 591 1‐km squares using ESRI ArcGIS 9.2 digital field mapping (ESRI, 2006). Surveyors comprehensively delineated and mapped each surveyed square, including any linear feature longer than 20 m. Detailed attribute information was recorded that allowed boundaries to be classified as either water, walls, fences, banks or woody features, and characterized, either by height or by shape. Full protocols and methodologies, including the field mapping handbook, are available at www.countrysidesurvey.org.uk.

As CS2007 data are complex including multiple features along a single field boundary (e.g., hedges, lines of trees, inland water, ditches, fences, and walls), initial results indicated that they yielded high degrees of nonmatches when compared to the generalized framework. To negate this issue, the CS data were generalized to single features, with woody linear features taking dominance in the hierarchy to match the model framework.

Although CS surveyors use OSMM digital lines to record their information, the simplified spatial framework of LCM 2007 meant that the line‐work of the two systems does not perfectly agree, despite visually appearing to be a good match. To remove this artifact, the areas around both the CS lines and the modelled linear network line‐work were spatially buffered by 5 m, and the intersecting area was used for the comparison. A point sample framework within this intersecting area at 5‐m intervals along each linear feature was then used to test the similarity between the modelled data and CS. Comparison between points within the intersected buffer of each framework was recorded for both sets of data (CS and model). Both sets of points were classified as either woody linear features (1) or other linear features (0) and were then compared using a nearest neighbor analysis. Finally, a kappa statistic (Cohen, 1960) was computed against the validation data for the 5‐m point interval classification which compares the accuracy of the system to the accuracy of a random system, and it is a general statistic that can be used for classification systems.

## 3 Results

**Figure 1:**
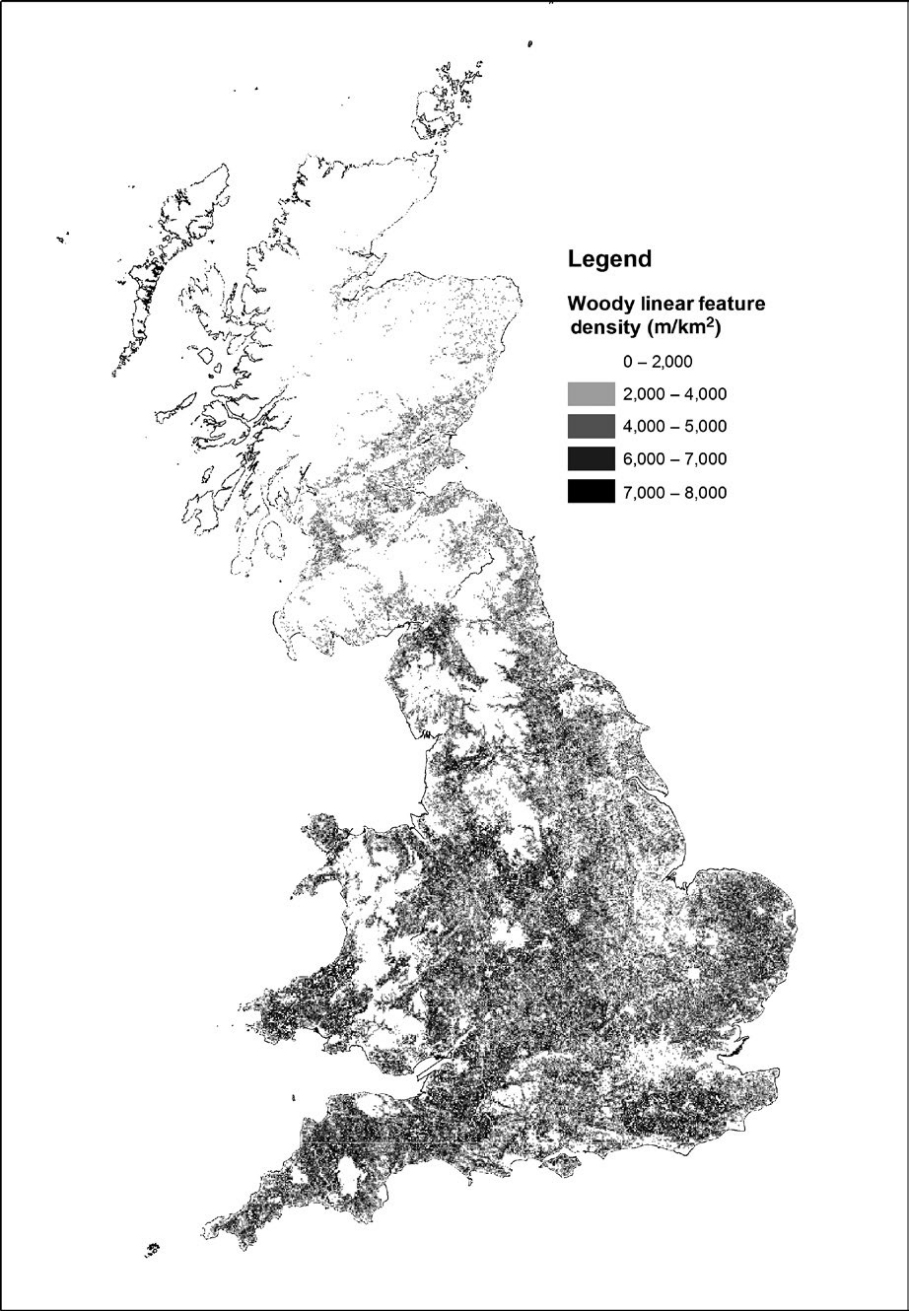
Woody linear feature density for GB estimated by the linear network model (m/km^2^)

**Figure 2:**
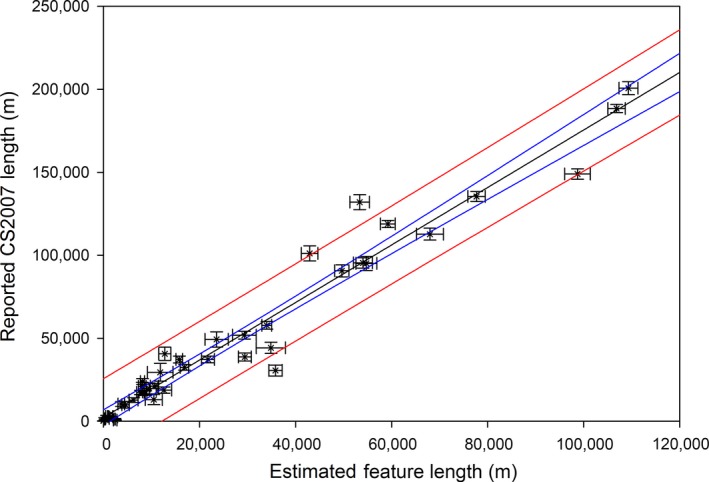
Linear regression of estimates of woody linear feature lengths for each ITE land class from CS2007 plotted against estimates from the model aggregated to ITE land classes (*r* ^2^ = .98), with confidence and prediction interval lines

For the large‐scale estimates, Figure 1 shows the model predictions for the density of woody linear features per km square in GB. The results indicate high densities toward the south of GB and much lower densities in the north. Table 1 shows the national statistical estimates of hedgerow length (by country) from the model and published from CS2007; the CS estimates are qualified by standard errors. Across GB and England, model estimates were around 60% of those generated directly from the CS 1‐km square samples although they were more similar in Scotland (73%) and less in Wales (51%). The estimates for the 45 land classes from the model, plotted against the CS estimates, are presented in Figure 2; there is good agreement (*r* ^2^ = .98), but the slope clearly shows that the model predicts fewer features than estimated from the CS sample by 43%.

**Table 1:** Comparison of national estimates of hedgerow length for GB and its component countries from the model and Countryside Survey 2007 (CS2007). CS2007 estimates are qualified by standard errors

Country	Total woody linear features (km × 10^3^)	
	Model	CS2007
Great Britain	420.9	700 ± 22.3
England	333.0	547 ± 20.1
Scotland	34.0	46 ± 5.5
Wales	53.8	106 ± 7.9

**Figure 3:**
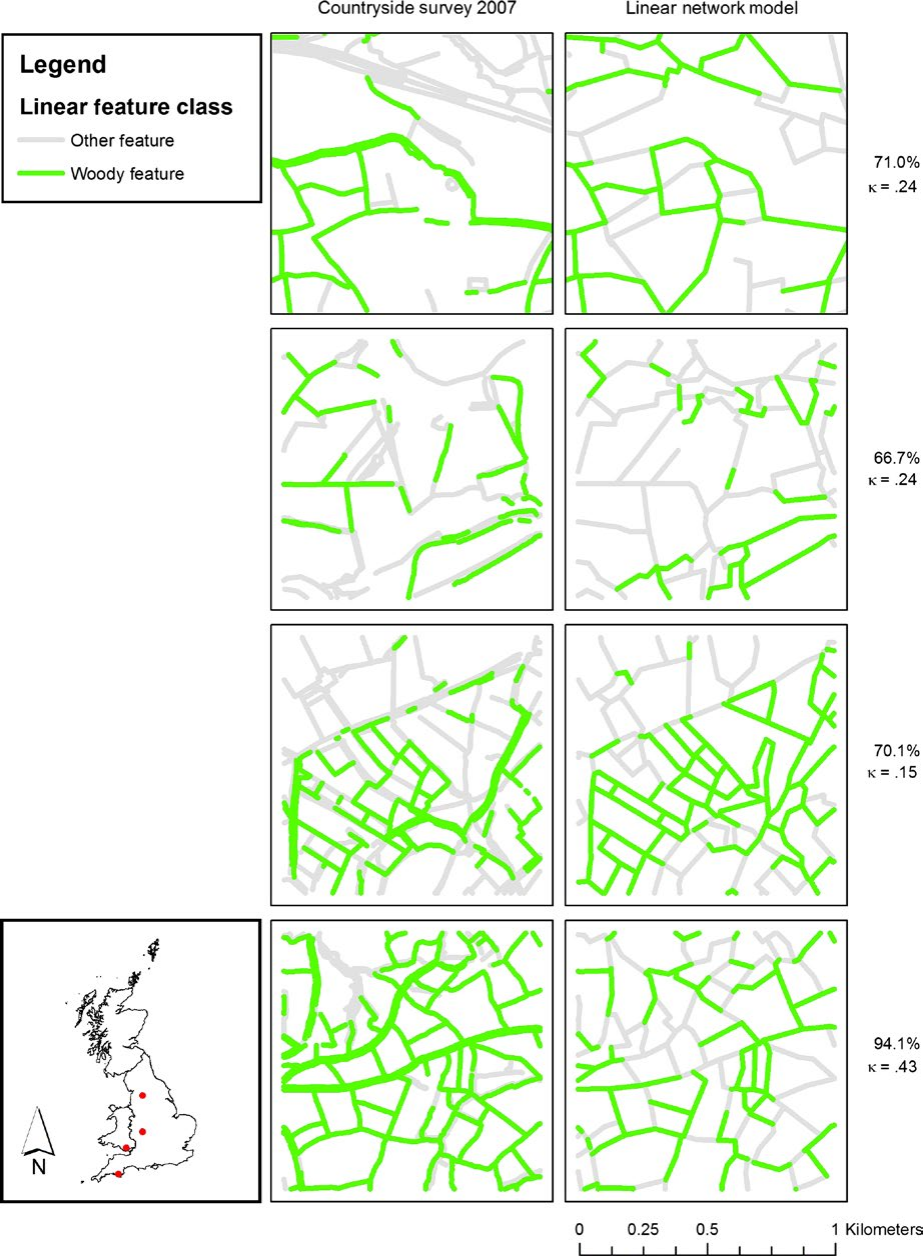
Actual and predicted extents and locations (proportions) of woody linear and “other” features for a sample of CS squares. Values are percent accuracy and kappa statistic for each comparison

A comparison of the modelled linear feature lengths and the lengths of linear features (both woody and other) as measured in the actual CS 1‐km squares as points are shown as summed totals in Table 2. The results indicate that the number of point matches between modelled and recorded lengths were higher than the number of nonmatches for both woody and “other” linear features. Examples of four survey squares showing the distribution of woody linear features and other species in CS and comparative predictions from the model are presented in Figure 3; the squares represent a range of agreement levels. The percentage accuracy associated with predictions for each square refers to the extent to which the predicted lengths of woody linear features accord with the actual locations (along a 5‐m spaced series of points) of woody linear features recorded in the field. In some squares, the predictions were poorer than for those shown in Figure 3, although the overall proportional accuracy of the model for both woody linear features and “other” linear features given in Table 2 shows that the majority of features were correctly predicted.

**Table 2:** Locational accuracy of the model predictions (mapped as linearly spaced points) within the intersected area of the CS2007 linear features and the modelled linear features. Figures represent total numbers of points for each matching and nonmatching (shaded) feature class

		Countryside Survey		
		Woody linear feature point	Other linear feature point	% agreement
Model	Woody linear feature point	109,854	80,623	58
	Other linear feature point	146,737	288,115	66

**Figure 4:**
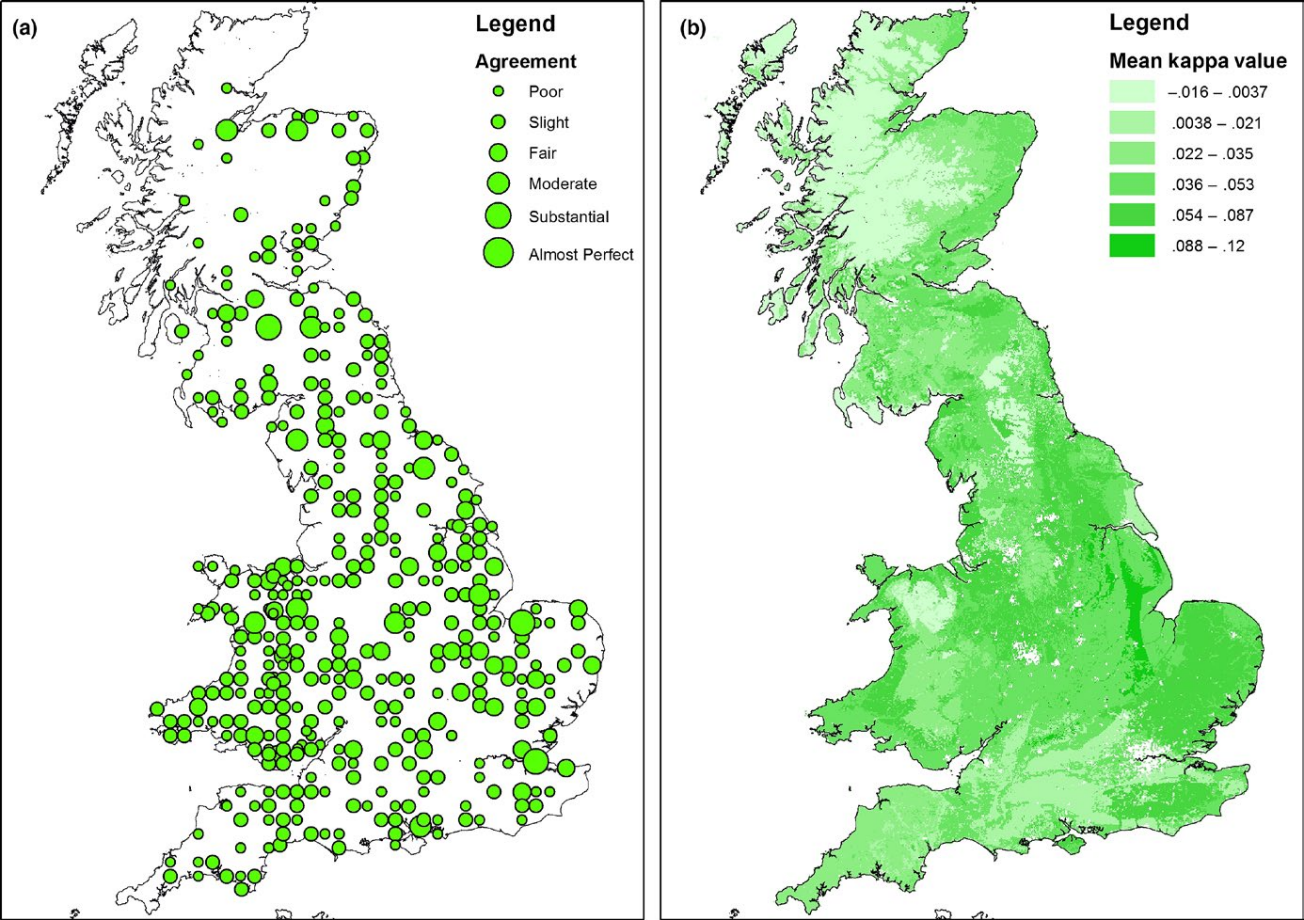
(a) GB map of CS survey square locations indicating Cohen's kappa coefficients for point classification accuracy of predictions for the location of woody linear features. (b) Modelled vs observed linear network agreement for CS survey squares mapped by land class for Cohen's kappa coefficients for point classification coefficients for point classification accuracy of predictions for the location of woody linear features

**Figure 5:**
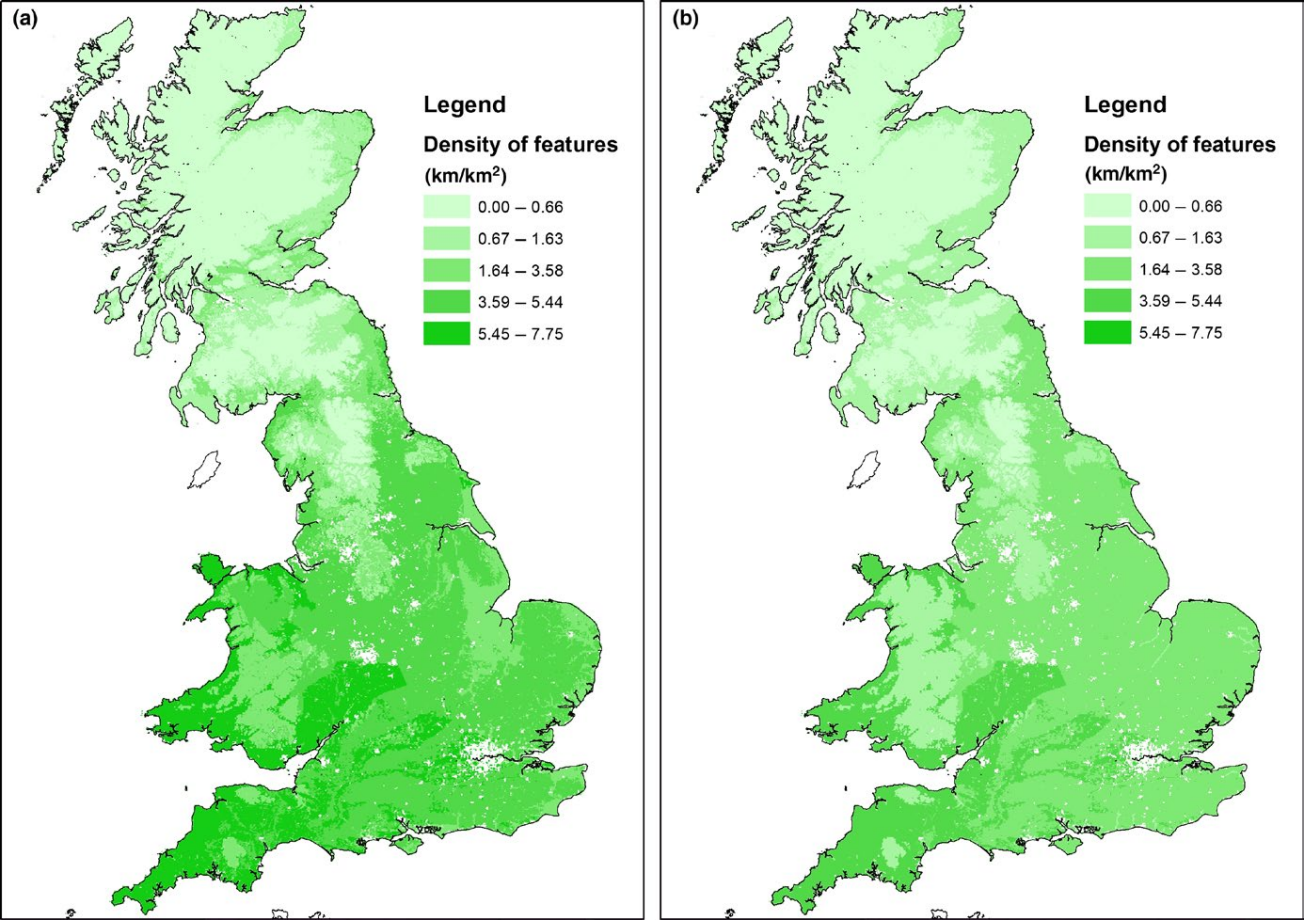
(a) Woody linear feature density (km/km^2^) from CS2007 field survey, mapped as land class means; b) modelled linear woody feature network density (km/km^2^) mapped as ITE land class means

The spatial accuracy of the classification is indicated in Figure 4 which shows Cohen's Kappa statistics for the point‐based comparisons between modelled and field‐recorded data. Figure 4a shows the results for all squares containing woody linear features, while Figure 4b shows the values averaged by land class. Kappa values are absent in areas where there are no recorded hedgerows. The levels of agreement vary between poor agreement and perfect agreement and do not appear to be spatially biased. Figure 5 compares the estimates of woody linear feature density based on CS2007 (5a) with the estimates based on the modelled linear framework (5b).

## 4 Discussion

The output of our study is a unique map describing the locations of, and classifying, individual linear boundary features at a national scale (Figure 1). The map was derived from a simple data‐led model that has created consistent categories and results across all regions. In addition broad classifications of linear feature types the model provides structural information on the features in the model which may be further interrogated in the future to improve the model and its uses.

The outputs of the model at a national scale are concordant with published statistics (Table 1) and spatially consistent with CS results (Figures 2 and 5). However, the estimates are generally on average 40% lower than those generated from the CS sample. Figure 3 indicates that the model errors are more commonly associated with the omission of hedges rather than identification of false hedges. The method of matching boundaries is not perfect, as the datasets being compared are independently derived and boundaries are often complexes of different features located very close to one another, which may include, for example, two hedges bounding another linear feature (such as a green road), or coincident lines of trees and hedges. Additionally, woody linear features in GB are highly variable dependent on individual hedge management practices, regional cultural norms, engagement with agri‐environment schemes etc. and may vary between a short (<1 m) and narrow (<1 m) feature resembling a wall and a wide unmanaged hedgerow between 5 and 10 m wide including standard trees with substantial crowns (Countryside Survey, 2007). The match appears to be better in the southwest of England, and this may be because the hedgerow areas are often earthen banks topped with gorse (*Ulex europaeus*).

A measure of confidence in the output, expressed as Kappa statistics, can be seen in Figure 4, showing results for both individual CS survey squares and land class means. There was no relationship between hedgerow length and confidence (i.e., the model is not better at predicting hedges where there are a lot of them), and in general, there is “fair” to “moderate” agreement.

To date, CS data have been used for hedgerow assessments to underpin national policy on their management (Norton et al., 2012). While Figure 2 shows the strong agreement between land class means for CS2007 and the model outputs (despite the offset axes), the maps in Figure 5 provide more spatially explicit information. While densities are again lower in the model outputs, the east–west polarization (5a; with higher densities of hedges in the west of the country), is less sharply divided in the map of modelled data (5b), with relatively more hedges being seen in East Anglia (land classes 2, 3, and 4). This may in part result from the greater complexities of landscapes in the south and west of GB with smaller fields and potentially more double boundaries which may not be successfully differentiated within the model, where they would be in the field by CS surveyors.

For national policy makers, the level of spatial disaggregation in CS, accompanied by detailed land class information on hedgerow condition provides valuable evidence for decision‐making, but for users requiring location‐specific information in order to make decisions, land class averages are inadequate. In the case of areal Broad Habitats, data surveyed in the field by CS have been supplemented by satellite‐derived LCMs (Morton et al., 2011) which provide coarser but more spatially comprehensive information at a national scale. Field survey data provide detail on habitat and landscape feature types and their condition alongside other variables not obtainable by satellite, but where information about individual parcels of land are required for specific locations, LCM2007 provides the most comprehensive data source. The linear model described here similarly provides coarse‐level information on linear features at a national scale. Britain's Ordnance Survey (OS) carries authority, and people have relied on its mapping skills for over a century. Currently, Ordnance Survey is currently developing their own woody linear feature layer under contract to the Rural Payments Agency who require the information in relation to farmer payments for maintenance and enhancement of features under Common Agricultural Policy (CAP). When available, these data which also use earth observation (EO) data will be compared to model outputs.

The model has a number of potential practical, scientific, and policy uses which are explored further here. These include its potential use as a methodology for administering rural payments relating to linear woody features (above). Although, as many payments commonly relate to the condition of features, further investigation of the measures attributed to each woody linear feature and their relevance to condition measures would need to be undertaken; furthermore, closer to 100% accuracy would be required for payment administration. CS data, potentially in combination with the linear model, would be appropriate for such an analysis. Other potential users of such data include the conservation sector (Wildlife Trusts, RSPB, biodiversity recorders, Local Nature Partnerships) who may want to enhance habitats/landscape structure/connectivity/biodiversity at local scales using woody linear features. Hedgerows are recognized as very significant component of GB landscapes and are currently recognized as habitats of principal importance under Section 41 of the Natural Environment and Rural Communities (NERC) Act in England and equivalent legislation affecting Scotland and Wales. This led to a number of regional field hedgerow mapping exercises being performed in order to take account of the extent and condition of hedgerow habitats. These exercises are resource intensive (even with the use of volunteers) and inconsistent in their coverage at a national scale due to their dependency on buy‐in at regional levels (although much effort was made to ensure consistency of recording). A new consistent national dataset adds greatly to existing regional data in providing a better understanding of their role in providing habitats for local biodiversity and connecting up semi‐natural habitats.

For potential business use, such as the development of hedges as a wood fuel resource (Wolton, 2014) for bioenergy, a dataset describing the woody linear network will be relevant to the identification of suitable locations for relevant infrastructure such as biomass generators and anaerobic digesters.

This dataset also enables us to increase our scientific understanding of landscapes and how they provide essential ecosystem services. Woody linear networks are a significant but, as yet, under accounted for component of landscapes which contrast greatly with the field/parcel vegetation with which they are associated. The ability to improve landscape models of ecosystem function by including woody linear features is likely to impact upon current estimates of what and how our landscapes deliver different ecosystem services. For example, studies on how landscapes impact on bird diversity have shown that including the detailed components of landscapes (including landscape features) gives us a much improved understanding of what factors affect bird presence (Rhodes, Henrys, Siriwardena, Whittingham, & Norton, 2015). Similarly, it is known that hedges influence a whole range of ecosystem services from disease spread to the provision of clean water or climate mitigation (as detailed in the introduction); this dataset provides the potential for accounting for that influence alongside that of land cover in parcels in models of ecosystem function.

During the construction and testing of the model, a number of different approaches were taken including the use of CS field data to train the model, but the simplest model consisting of a simple query of the height characteristics proved to be both the most effective and robust. Detailed CS data might potentially provide a valuable dataset for understanding the performance of the model and thereby improving it, but this would require a significant amount of time and resource and may only serve to highlight issues around the resolution and spatial accuracy of the spatial framework and the CS dataset. Options for improving the model in the future using national‐scale data include the potential use of land cover information about the land parcels on either side of the boundary, which may be correlated with the linear feature type (this could be verified using CS sample square data). LiDAR data are also a potential data source for improving the product, and has been used in the development of regional models of woody linear features (Bailly, Lagacherie, Millier, Puech, & Kosuth, 2008; Ferraccioli et al., 2014). It has not however yet (to the authors’ knowledge) been collected or interpreted in a consistent way across GB.

Another possibility may be to use citizen science to further validate and improve the quality of information, including historical data collected as part of the regional hedgerow mapping exercises described above, although this would need to be carried out using a strategic and consistent framework to ensure the consistency and quality of the data. Potential approaches include placing the data on the internet for users to validate. The approach could be linked to temporal reviews and revisions to provide statistics of hedgerow change. This could, for example, help to target particular management approaches including restoration or recreation of woody linear features under agri‐environment schemes.

Given the lack of any such product currently, the information it provides is valuable and although incomplete, the hedge model is generally accurate. Clearly, for users at local levels, there is a great opportunity (potentially through the development of appropriate software) to supplement the model data with new or more accurate data collected at local scales either through volunteer approaches on the ground or the addition of regional government data (where available). The value of a national model is consistency of approach and as stated above, any enhancements to the model outputs at local levels should endeavor to retain and build on this consistency.

In conclusion, the information presented here offers great potential to further the management and conservation of hedgerows in GB, improve delivery of ecosystem services, and to improve landscape resilience, and the approach described is an easily translatable model that can be applied in different parts of the world given the availability of appropriate data.

## Conflict of Interest

None declared.
